# George B. Harriman, D.D.S.

**Published:** 1905-12

**Authors:** 


### GEORGE B. HARRIMAN, D.D.S.

Died, at Moosehead, Maine, May 22d, 1905, of pneumonia, George B. Harriman, D.D.S., of Boston, Mass.

George B. Harriman was born at Groton, New Hampshire, on March 18th, 1837. He received his preliminary education at New Hampton Institute, studied dentistry under John Clough, M.D., of Boston, in 1857, and entered practice in 1858.

After several years of practice he entered Boston Dental College as a student and graduated in 1870. The next year he was elected Dean of the College, in which capacity he served for two years; later he was chosen as Trustee and remained a member of that Board until after the school was transferred to the Tufts College Corporation.

As a student of the College he became deeply interested in microscopy under Professor Rufus King Brown and for several years was an enthusiastic student in that science, conducting certain important investigations. For a time he was Professor of Histology and Microscopy at the College.

Dr. Harriman was one of the eight graduates who met in one of the lecture rooms of the old College at No. 5 Hamilton Place on March 4th, 1872, and founded this association. He was chairman of the Executive Committee during the first two years of its existence, was President in 1897, again chairman of the Executive Committee in 1898, and an active member until his death.

For a long time he had suffered from asthma and had gradually withdrawn in part from active practice. Last March he closed his office on Park Street and went to California, returning early in May. It had been his custom for several years to visit the Moosehead region on the opening of the fishing season in the Spring; and though urged by friends not to do so this year, he went to Moosehead Lake on May 13th, accompanied by Mrs. Harriman. There pneumonia supervened, and he died on the 22d. As has been beautifully said, "The Call of the Wild led him back close to Nature's heart, and with his head on her bosom, he slept."

Dr. Harriman was a prominent Freemason, a life member of Boston Commandery, an earnest supporter of the First Free Baptist Church, of Roxbury, and though he never aspired to salaried political office he was at one time active in Republican politics, serving as a member of the State Central Committee. He was a man of moderate wealth, of sterling character and Christian brotherhood. He leaves a widow, his second wife, and three sons.

In respect to his memory, the Boston and Tufts Dental Alumni Association adopted the following resolutions on October 11th:

WHEREAS, the hand of Providence has removed from us our honored member and colleague, Dr. George B. Harriman; and

WHEREAS, in his decease we have lost one of the founders of our association, who in committee, as President, and as an active member until his death, evinced a warm interest in its welfare; who as Dean, as Professor, and as Trustee of our Alma Mater gave to her freely of his wisdom; and who in many years of practice set before us a worthy example of fidelity to his patients, of fairness toward his colleagues, and of kindly interest in his younger professional brethren; therefore, be it

*Resolved,* that we desire to express to the bereaved family our sympathy and sorrow in their affliction, and our admiration for the professional and personal qualities of our colleague; and

*Resolved,* that these resolutions be spread upon the records of our association, a copy sent to the family of our departed brother, and others to the dental journals for publication.

FRED'K S. FOGG,

E. W. BRANIGAN,

JAMES R. PIPER,

*Committee.*

MARION L. WOODWARD,
*Recording Secretary,*
*Boston and Tufts Dental Alumni Association.*

